# Preoperative hospital length of stay as a modifiable risk factor for mediastinitis after cardiac surgery

**DOI:** 10.1186/1749-8090-8-45

**Published:** 2013-03-12

**Authors:** Stephane Leung Wai Sang, Rakesh Chaturvedi, Ahsan Alam, Gordan Samoukovic, Benoit de Varennes, Kevin Lachapelle

**Affiliations:** 1 Divisions of Cardiac Surgery, Royal Victoria Hospital, McGill University Health Center, 687 Pine Ave. West, Room S 8.73, Quebec, H3A 1A3, Montreal, Canada; 2 Divisions of Medicine, Royal Victoria Hospital, McGill University Health Center, 687 Pine Ave. West, Quebec, Montreal, H3A 1A3, Canada

## Abstract

**Background:**

As high-risk cardiac patients frequently remain within hospital while waiting for surgery, the aim of the present study was to determine the role of preoperative length of hospital stay on mediastinitis, and also, to assess contemporary risk factors for this complication.

**Methods:**

The source population consisted of 6653 consecutive patients undergoing coronary bypass surgery, valve surgery, or both between September 2000 and September 2009 at a single tertiary care hospital. A retrospective cohort analysis was used to assess the effect of 18 preoperative variables, including length of stay, on mediastinitis.

**Results:**

Mediastinitis developed in 108 patients (1.6%) resulting in an in-hospital mortality rate of 13.9%. Independent predictors of mediastinitis included obesity (2.59, CI 1.58-4.23), COPD (2.44, CI 1.55-3.84), diabetes (2.16, CI 1.44-3.24), and impaired estimated glomerular filtration rate. Preoperative hospital stay was also found to be an independent risk factor leading to a 15% increased risk of mediastinitis per week of stay. The primary wound pathogen was coagulase negative *staphylococcus* (82%) followed by multi-flora isolates (49%), but was unrelated to hospital stay.

**Conclusions:**

In addition to the traditional risk factors, prolonged preoperative hospital stay is also a significant and potentially modifiable predictor for the development of mediastinitis following cardiac surgery. All efforts should be made to minimize the delay in operating on hospitalized patients awaiting heart surgery.

## Background

Despite improved surgical techniques and preoperative antibiotic prophylaxis, surgical site infections, more specifically mediastinitis, remains a potentially fatal complication after open-heart surgery with an incidence of 0.6% - 2.65% [1-5]. In-hospital mortality ranges from 14 to 23%, even when mediastinitis is correctly treated [6,7], and it has been shown to have a significant impact on hospital costs [8].

Well-described risk factors for mediastinitis include diabetes mellitus, chronic obstructive pulmonary disease (COPD), obesity, as well as harvesting of bilateral internal mammary arteries [9-12]. In many Canadian Provinces, patients too precarious to wait at home, often stay in hospital for an extended period before surgery can be performed. There are many reasons for this situation, which may include restricted operating room access, lack of ICU beds, or delays due to more urgent cases [13]. Knowing that hospital stay may predispose a patient to skin colonization with more virulent hospital-based pathogens, we hypothesized that a prolonged preoperative hospital stay would also result in an increased incidence of mediastinitis. The consequences of prolonged preoperative hospital stay on cardiac surgical outcome have not been previously studied.

The aim of the present study, therefore, was to identify preoperative risk factors for mediastinitis in current practice, and to assess the role of preoperative length of stay on deep sternal wound infection in the context of increasing surgical delays.

## Methods

### Study population and study design

The study population comprised of 6653 consecutive adult patients undergoing median sternotomy during the period between September 2000 and September 2009 at a single tertiary care hospital in Montreal, Canada. Heart transplant patients, adult congenital and patients receiving ventricular assist devices were excluded from this cohort.

Preoperative variables that were collected prospectively as part of a clinical registry maintained by the Division of Cardiac Surgery included the following: age, sex, obesity, ejection fraction, type of operation (coronary, valve, or combined procedures), smoking, diabetes mellitus, COPD, chronic renal failure (CRF), preoperative creatinine, preoperative estimated glomerular filtration rate (eGFR), congestive heart failure (CHF), hypertension, redo surgery, left main coronary disease, preoperative hospital length of stay, operative status (elective, urgent, or emergent), and Parsonnet score. Any missing data was obtained by reviewing individual patient charts at the time of the study. This study was approved by the McGill University Health Center Research Ethics Board.

### Definitions

Mediastinitis was defined according to the Center for Disease Control and Prevention criteria [14]; however, all suspected cases were also confirmed by CT scan imaging of the chest. Superficial wound infections sparing the sternum were not classified as mediastinitis. Estimated glomerular filtration rate was derived using the CKD-Epi study equation using the preoperative serum creatinine value closest to the date of surgery [15]. Preoperative hospital length of stay was calculated as the number of days from hospital admission (under any service) until the day of surgery. The calculation of Parsonnet score has been previously described [16]. Obesity was defined as BMI greater than 30 kg/m^2^. Operative status was categorized as: emergent if the patient’s cardiac disease dictated that surgery be performed within hours outside of regular operating hours; urgent if the patient’s cardiac disease required the patient to remain in the hospital until the operation was performed; and elective if the patient’s clinical status allowed discharge from the hospital and readmission at a later date [17].

### Infection prophylaxis and operative technique

All patients receive a 2% chlorhexidine gluconate shower and mupirocin 2% ointment to both nostrils the night prior to surgery as well as the morning of. Antibiotic prophylaxis is given as Cefazolin 2 grams intravenously 30 minutes before skin incision and again after 4 hours. Additional doses are given every 4 hours, if required, until termination of the surgery. Patients undergoing valvular surgery, have a penicillin allergy, or who are methicillin-resistant *Staphylococcus aureus* (MRSA) positive receive Vancomycin 1 gram intravenously 30 minutes before skin incision. Removal of excess hair at the sternum, groin, and appropriate leg is performed with clippers in the operating suite. All patients are disinfected with Baxedine^©^ (Chlorhexidine 2% w/ 70% V/V alcohol solution) in the operating room prior to incision.

Cardiopulmonary bypass was performed using non-pulsatile flow. Cold blood cardioplegia was used to induce and maintain cardioplegic arrest. Patients are kept at mild hypothermia throughout cardiopulmonary bypass (lowest nasopharyngeal temperature 32°C). The left internal mammary artery, when required as per discretion of the surgeon, was harvested as a pedicle using electrocautery in all cases. Sternal closure was performed using seven or eight No.5 stainless steel wires. In the immediate 24 hr postoperative period, an insulin infusion titrated to a blood glucose defined by the intensive care physician was administered if blood glucose levels exceed 10 mmol/L. The insulin infusion was discontinued once the patient was transferred to the cardiac surgical intermediate care unit.

### Statistical analysis

Patient characteristics are expressed as proportions, means and standard deviations, or medians and interquartile ranges, as appropriate. A natural log transformation was made to Parsonnet score, since its distribution was highly skewed.

Logistic regression was used to examine the association of various demographic, comorbidity, and preoperative laboratory variables with mediastinitis. Models were adjusted for covariates that were significant at the univariate level. The Hosmer Lameshow Goodness-of-fit test was used to assess calibration of the logistic regression model, by assessing the observed and predicted event rates in subgroups (deciles) of the model population. A p-value threshold of 0.05 was used to determine statistical significance. All analyses were carried out using SAS version 9.1 (SAS Institute Inc., Cary, NC).

## Results

### Patient characteristics and incidence of mediastinitis

During the 9-year period from September 2000 to September 2009, 6653 patients underwent cardiac surgery resulting in 108 (1.6%) cases of mediastinitis. Of these, 87 included coronary artery bypass grafting surgery (CABG) with use of a single internal mammary artery, 16 included CABG without any mammary artery, while 5 were isolated valve cases. The overall distribution of operations included isolated CABG at 64.9%, isolated valve in 15.4% of cases, and combined valve and CABG in 14.9% of cases.

Table 1 describes the baseline characteristics of the study population. The mean age of the patients was 66.1 years with the majority having a systolic ejection fraction of more than 50%. The mean Parsonnet score was 18.7 ± 13.3 leading to a mean predicted mortality risk of 7.6%.

**Table 1:** **Baseline preoperative characteristics**^**a**^

**Variable**	**All patients N=6653**
Age, years (SD)	66 (10.9)
Male Sex, n (%)	4647 (69.9)
Obesity (BMI > 30 kg/m^2^), n (%)	461 (6.9)
% EF	
>50%, n (%)	3410 (60.6)
30-50%, n (%)	1499 (26.7)
<30%, n (%)	715 (12.7)
Type of Surgery	
CABG only, n (%)	4319 (64.9)
Combined valve + CABG, n (%)	986 (14.9)
Valve only, n (%) (Heart transplant, congenital, and ventricular assist devices excluded)	1024 (15.4)
Smoking (current/<1 year abstinent), n (%)	85 (1.3)
Diabetes, n (%)	1642 (24.7)
COPD, n (%)	703 (10.6)
CRF, n (%)	644 (9.7)
Mean preoperative creatinine, μmol/L (SD)	111 (73)
Mean eGFR, ml/min/1.73 m^2^, (SD)	65 (22)
CHF, n (%)	2458 (37.0)
Hypertension, n (%)	4216 (63.4)
Redo surgery, n (%)	499 (7.5)
Left main disease, n (%)	1018 (15.3)
Duration of Preoperative Hospital Stay, median (IQR)	2 (2–3)
Emergency surgery, n (%)	571 (8.6)
Parsonnet score, mean (SD)	18.7 (13.3)

^a^ For definitions please refer to reference 16.

*CHF* congestive heart failure, *COPD* chronic obstructive pulmonary disease, *CRF* chronic renal failure, *EF* ejection fraction, *eGFR* estimated glomerular filtration rate, *IQR* interquartile range, *SD* standard deviation.

### Risk factors for mediastinitis

Preoperative predictors for mediastinitis are shown in Table 2. Univariate risk factors were advanced age, Parsonnet score, CHF, COPD, diabetes, hypertension, left main disease, peripheral vascular disease, obesity, decreasing eGFR, operative status, and prolonged preoperative length of stay. After adjusting for all significant factors in univariate analyses only obesity, COPD, diabetes, preoperative length of stay, and decreasing eGFR were independent predictors of mediastinitis, with obesity being the most significant (OR 2.59, 95% CI 1.58-4.23). Standard eGFR cut-points of 30 and 60 ml/min/1.73 m^2^ were used in Table 2; however, there was no eGFR cut-off value above which the risk of mediastinitis became significant, since the relationship between both elements remained linear across all eGFR values (data not shown). The area under the receiver operating characteristic curve for our fully adjusted model was 0.734. This implies that our model, which uses easy to collect variables, is acceptable to accurately discriminate those at risk of mediastinitis after cardiac surgery. The Hosmer Lameshow Goodness-of-Fit test also showed good calibration of our model (Chi-square 10.48, degrees of freedom=6, p-value=0.11).

**Table 2:** Preoperative factors associated with mediastinitis based on cohort analysis

**Covariate**	**Univariate OR (95% CI)**	Adjusted OR (95% CI)*
Age (per 10 years)	1.28 (1.06-1.53)	NS
Parsonnet Score (per ln)	1.80 (1.34-2.40)	NS
CHF	1.53 (1.04-2.25)	NS
COPD	3.44 (2.24-5.30)	2.44 (1.55-3.84)
Diabetes	2.89 (1.96-4.24)	2.16 (1.44-3.24)
Hypertension	2.68 (1.63-4.41)	NS
Left Main Disease	1.63 (1.03-2.59)	NS
PVD	2.72 (1.64-4.50)	NS
Obesity	4.33 (2.74-6.85)	2.59 (1.58-4.23)
Estimated GFR (ml/min/1.73 m^2^)		
≥60	Reference	Reference
30-59	1.86 (1.20-2.90)	1.56 (1.00-2.45)
<30	3.53 (2.12-5.87)	2.41 (1.42-4.09)
Operative Status:		
Urgent vs elective	2.20 (1.40-3.46)	NS
Emergent vs elective	1.44 (0.65-3.22)	NS
Preoperative length of stay (per week)	1.17 (1.05-1.31)	1.15 (1.02-1.31)

^*^ Adjusted model includes all variables significant at the univariate level. Hosemer-Lameshow Goodness-of-Fit Test Chi-square=10.48, df=6, p-value=0.11.

*CHF* congestive heart failure, *COPD* chronic obstructive pulmonary disease, *ln* natural log, *OR* odds ratio, *PVD* peripheral vascular disease, *NS* non-significant.

Furthermore, a dose–response relationship was found with regards to preoperative hospital stay, whereby a stay beyond 7 days was associated with a 2.43-fold increased risk of mediastinitis compared to a preoperative stay of 4–7 days which increased this risk by 1.39 times (Table 3).

**Table 3:** Incidence and risk of mediastinitis according to preoperative length of stay

**Covariate**	**N**	**Cases of mediastinitis (%)**	**Adjusted OR (95% CI)**
Preoperative length of stay:			
0-3 days	5080	71 (1.4)	Reference
4-7 days	876	17 (1.9)	1.39 (0.81-2.37)
>7days	589	20 (3.3)	2.43 (1.47-4.02)

*OR* odds ratio.

### In-hospital mortality and management of mediastinitis

There were 381 in-hospital deaths. The in-hospital actuarial mortality rate for cardiac surgery cases complicated by mediastinitis was 13.9% compared to 5.6% in those patients without mediastinitis (OR 2.72, 95% CI 1.56 - 4.75).

Management of patients with mediastinitis was either by non-operative or operative means. Twenty-eight patients who had a stable clinical picture were treated by conservative management involving vacuum-assisted closure therapy with intravenous antibiotics. When the overall clinical picture displayed evidence of deterioration due to unremitting infection or lack of improvement secondary to an unstable sternum, operative revision was performed. Sternal debridement and re-wiring was utilized in 16 patients, while sternal debridement with pectoralis muscle flap advancement was performed in 64 cases of mediastinitis.

### Bacteriology

Of the 108 cases of postoperative mediastinitis, no bacteriology could be found in 4 of the cases after chart review. In the remaining 104 cases, the most common pathogen was coagulase negative Staphylococcus (82%) followed by Enterococcus (22%). Mixed flora was found in 49% of cases of mediastinitis (Table 4). There was no significant relationship between bacterial pathogen and preoperative hospital stay.

**Table 4:** Prevalence of microorganisms isolated from sternal wounds in cases of mediastinitis

**Microorganism**	**Frequency (%)**
Coagulase-negative *staphylococcus*	82
*Enterococcus*	22
*Escherichia coli*	11
*Klebsiella*	11
*Staphylococcus aureus*	9
*Candida albicans*	9
*Corynebacterium*	7
Other	26
Mixed flora	49

## Discussion

Although cardiac surgery is currently undergoing innovative changes in its methods of exposure to allow less invasive approaches to valve and coronary surgery, the standard median sternotomy has not changed since its inception in 1957 [18]. Although the complication of mediastinitis is rare, it continues to be a significant source of morbidity, mortality, and cost. The incidence of mediastinitis in this contemporary patient population was 1.6%, which is consistent with the range of 0.6%- 2.65% reported in studies performed within the last decade [1-5]. Our in-hospital actuarial mortality rate of 13.9% is on the lower range of the 15-20%, which has been observed previously [3,4]. In contrast to previous studies, we did not limit our analysis to CABG procedures only, but included combined CABG and valve, as well as valve only. The procedure type was not associated with mediastinitis (data not shown).

In accordance with other studies our analysis found that diabetes [6,10], COPD [2,19], impaired preoperative kidney function [4,20], and obesity [2,4,6,9,12] to be independent risk factors for mediastinitis. The mechanism by which diabetes leads to mediastinitis is unclear. There is evidence relating cardiac surgical wound infections with perioperative hyperglycemia [21,22]. Such elevated glucose levels have been found to be associated with a pro-inflammatory process leading to increased susceptibility to infection as well as organ dysfunction [22]. In this study, COPD increased the risk of mediastinitis by 2.4 folds. The coughing induced by COPD is hypothesized to lead to sternal instability, therefore, increasing the risk of bacterial translocation and hence, sternal infection [23]. Previous studies have related preoperative renal failure on dialysis with deep sternal wound infections following cardiac surgery [4,20]. In this study, we used eGFR as a determinant of renal function, as this measurement is more accurate than serum creatinine alone. Our results illustrate that even impaired preoperative kidney function is an independent predictor for the complication. Our cohort analysis, as in others, also found obesity to be the most significant risk factor for deep sternal wound infections. According to Milano et al. the fixed prophylactic antibiotic doses used in adult surgery may be inadequate given the large volumes of distribution in the obese [24]. Another plausible mechanism includes the large lateral forces on the sternum pulling the sternal halves apart and predisposing to sternal dehiscence.

This study demonstrates that extended preoperative hospital stay is a significant risk factor for mediastinitis by both univariate and adjusted models. Each week of hospital stay preoperatively was associated with a 15% increased risk of mediastinitis. This is a novel and also potentially modifiable risk factor. To our knowledge only one previous study revealed that the interval between hospital admission and operation to be a risk factor for deep sternal wound infection [25]. Conklin et al. also observed that a preoperative hospital length of stay more than 7 days was associated with a 4.4-fold increased risk of overall surgical site infections after cardiac surgery [26]. Although a prolonged preoperative stay could lead to colonization of antibiotic-resistant nosocomial pathogens, our analysis revealed no significant relationship in the distribution of bacteria found on culture with the operative status or preoperative length of stay. Perhaps, a more plausible explanation is that in-patients are often kept fasting while awaiting urgent surgery, and therefore, may be nutritionally deficient in the perioperative period. It is well established that adequate caloric intake is necessary for proper wound healing. Hennessey et al. found that hypoalbuminemia was an independent risk factor for surgical site infections after gastrointestinal surgery, and that an albumin less than 30 mg/dL was associated with deeper infections [27]. As a result, patients with critical coronary disease awaiting urgent surgery in the hospital could be predisposed to mediastinitis based on their nutritional status.

Our results clearly indicate a need to reduce preoperative wait-times for cardiac surgery. Efforts such as delaying elective cases to prioritize in-hospital patients, as well as, discharging patients well enough to go home may help to minimize this risk factor.

As for the bacteriologic analysis, the most commonly isolated sternal pathogen comprised of coagulase-negative *staphylococcus* while *Staphylococcus aureus* was present in only 9% of wound cultures. Moreover, 49% of mediastinitis cases grew multiple organisms. These results differ from recent studies where *S. aureus* was the predominant bacteria present in 32-50% of isolates with coagulase negative staphylococcus present in 19-24% of cases [1,2,5]. Although it is possible that the unusually high frequency of coagulase negative staphylococcus is due to cutaneous contamination, this result helps to clarify our lower than expected hospital mortality rate of mediastinitis since surgical site infections from *S. epidermidis* present a more indolent course. More importantly, this analysis brings into question the effectiveness of using 1st generation cephalosporins as antibiotic prophylaxis in cardiac surgery since they do not adequately guard against coagulase negative *staphylococcus*. According to the Society of Thoracic Surgeons practice guidelines [28], Class I recommendations support use of a beta-lactam antibiotic, more commonly Cefazolin, as first-line for standard cardiac surgical prophylaxis where the incidence of MRSA is low. This has been our routine practice. Given the high rate of *S. epidermidis* in sternal wound infections demonstrated in the current study, we therefore suggest the use of Vancomycin as surgical prophylaxis in patients with a preoperative hospital stay beyond 3 days since they are at greater risk of deep sternal wound infections.

### Limitations

There are several limitations to our study. First, it is a retrospective study and as such, the results may be affected by confounding variables not controlled for in the study. In addition, there was no pre-specified follow-up of patients to assess wound infection following surgery, which could allow for patients with delayed mediastinitis to be lost to follow-up. However, as a tertiary care center, the majority of patients presenting at a peripheral hospital with a potential surgical complication are referred back to our institution. Currently, our database does not contain cardiopulmonary bypass and aortic cross clamp times for all patients, thus we could not evaluate its impact on mediastinitis risk. Another limitation is the failure of our database to capture the cause for the prolonged preoperative hospital stay. This is most often multifactorial. As our institution is a referral center, patients often come from afar, and therefore, remain in hospital until they can be operated on. The limited number of ICU beds in a public healthcare system also prevents prompt surgical intervention, thereby, further prolonging the hospital stay. For example, performing an emergency cardiac operation overnight often results in cancellations in the operating schedule the following day. Moreover, it is possible that patients remaining in hospital awaiting surgery did so because of an impaired medical condition (pneumonia, sepsis, CHF, etc.) that was not captured by our analysis. However, our study found no relationship between higher preoperative mortality risk as indicated by the Parsonnet score with the risk of mediastinitis. Lastly, this study was performed at a single institution, which may limit generalizability. However, this same circumstance also ensures that all patients were subjected to the same pre- and postoperative variables that could affect infection.

## Conclusions

The present data demonstrates that in addition to the traditional risk factors for mediastinitis, a prolonged preoperative hospital length of stay is also an important predictor of deep sternal wound infections. Minimizing the delay for hospitalized patients awaiting cardiac surgery, as well as optimizing the prophylactic antibiotic regimen in these patients should be further explored, as it likely represents a modifiable risk factor for mediastinitis.

## Abbreviations

CABG: Coronary artery bypass grafting; CHF: Congestive heart failure; COPD: Chronic obstructive pulmonary disease; CRF: Chronic renal failure; EF: Ejection fraction; eGFR: Estimated glomerular filtration rate; IMA: Internal mammary artery; IQR: Interquartile range; ln: Natural log; NS: Not significant; OR: Odds ratio; PVD: Peripheral vascular disease

## Competing interests

There were no competing interests in this study.

## Authors’ contributions

SLWS and GS helped to conceptualize and design the study. SLWS, RC and KL was involved in the data interpretation and analysis. SLWS drafted the manuscript. RC and SLWS examined the patient files as well as transferred the data from the departmental database to an Excel sheet. AA performed all of the statistical analysis and helped in the interpretation of data. BDV and KL designed the database and critically revised the manuscript. All authors read and approved the final manuscript.
